# Placenta models for HIV-1-ART-nicotine comorbidity research: advances and remaining challenges

**DOI:** 10.1038/s41420-026-03249-x

**Published:** 2026-07-13

**Authors:** Sandip Godse, Aditya N. Bade

**Affiliations:** 1 Department of Pharmacology and Experimental Neuroscience, University of Nebraska Medical Center, Omaha, NE 68198 USA; 2 Research Center for Developmental Toxicology and Pharmacology, University of Nebraska Medical Center, Omaha, NE 68198 USA

**Keywords:** Disease model, Experimental models of disease

## Abstract

Worldwide implementation of antiretroviral therapy (ART) for pregnant women living with human immunodeficiency virus type-1 (HIV-1) has markedly reduced mother-to-child HIV-1 transmission rate to less than one percent. Despite this benefit, adverse pregnancy outcomes remain a major concern. Placental deficits induced by antiretroviral drugs (ARVs) are increasingly recognized as a potential causative factor. Additionally, comorbid effects of substance use on placenta cannot be overstated. Addictive substances are commonly used by people living with HIV-1 (PLWH). However, globally, nicotine exposure through smoking or tobacco use remains a major comorbidity in PLWH. Yet, to date, whether and how, HIV-1, ARVs, and nicotine together exacerbate adverse effects on placenta and affect birth outcomes is unknown. Due to ethical concerns and impracticality of obtaining placenta tissues at different stages of pregnancy, appropriate experimental models that closely recapitulate in vivo placenta physiology are essential to define how HIV-1, ART, and nicotine independently or collectively affect placental development and functions. Herein, we provide a comprehensive review of the experimental placenta models that have been or are yet to be utilized to study placental comorbid pathobiology induced by HIV-1 infection, ART, and/or nicotine exposure. We emphasize how each model is used (or underused) to interrogate infection biology, pharmacokinetic profile, and toxico-pharmacologic mechanisms. Moreover, key limitations associated with each placenta model and necessary improvements for investigating HIV-1 and associated comorbidity are highlighted. Overall, this review seeks to educate both clinical and basic scientists on advances made, and remaining challenges in understanding comorbid effects of HIV-1-ART-nicotine on pregnancy outcomes using experimental placenta models.

## Facts

- Antiretroviral therapy (ART) during pregnancy and breastfeeding significantly reduced risk of mother-to-child HIV-1 transmission, however adverse pregnancy outcomes remain a major public health concern.
- Nicotine exposure through smoking or tobacco use is a major substance use in pregnant women living with HIV-1.
- The placenta is central to HIV-1 and associated comorbidity-linked adverse pregnancy outcomes.
- While in vitro cell culture systems, ex vivo human placenta explants, and animal models have substantially advanced our understanding of independent effects of HIV-1, ART, or nicotine on placental development and function, the impact of combined exposures on placental physiology remains largely unexplored.
- Improvement in placenta models is needed for studying HIV-1-ART-nicotine comorbidity linked infection biology, pharmacokinetic profile, and toxico-pharmacologic mechanisms at maternal-fetal interface.

## Open Questions

- How does co-exposure of HIV-1, ART, and nicotine influence placental development and function?
- What are the challenges with the application of commonly utilized experimental placenta models in investigating comorbid effects of HIV-1, ART, and nicotine?
- What improvements in placenta models are needed to advance rigor and reproducibility in determining HIV-1 and associated comorbidity?

## Introduction

The recommendation of life-long antiretroviral therapy (ART) to all individuals living with human immunodeficiency virus type-1 (LWH), including pregnant and breastfeeding women, has drastically reduced the rate of vertical mother-to-child HIV-1 transmission [1, 2]. In the pre-ART era, the vertical HIV-1 transmission rate was up to 40%. However, ART has significantly reduced this transmission rate to less than 1% [3–5]. Due to the improved availability of antiretroviral drugs (ARVs) at an affordable cost worldwide, more than 80% of pregnant women living with HIV-1 (PWLWH) now have access to ART [6]. Consequently, the proportion of PWLWH who conceive on ART is steadily rising. The population of HIV-1-exposed uninfected (HEU) children born to PWLWH has now reached nearly 16 million, with more than one million children joining this group each year worldwide [7–11]. Despite significant gains in preventing perinatal HIV-1 transmission, risks of adverse events on maternal health and fetal growth remain a public health concern. Studies showed that HEU children are at elevated risk of preterm delivery, infectious morbidity, immune abnormalities, impaired growth, and neurodevelopmental deficits [10–16]. Numerous maternal health abnormalities are recognized as underlying causes for adverse fetal outcomes. These include maternal infection, persistent inflammation, and metabolic disorders. However, placental deficiencies remain a major underlying cause [17–20]. The placenta is not only a key regulator of fetal development but also determinant of long-term postnatal outcomes. Yet effects of HIV-1 and related comorbidities on placental development and functions and its linkage to fetal developmental outcomes remain understudied.

**Fig. 1:**
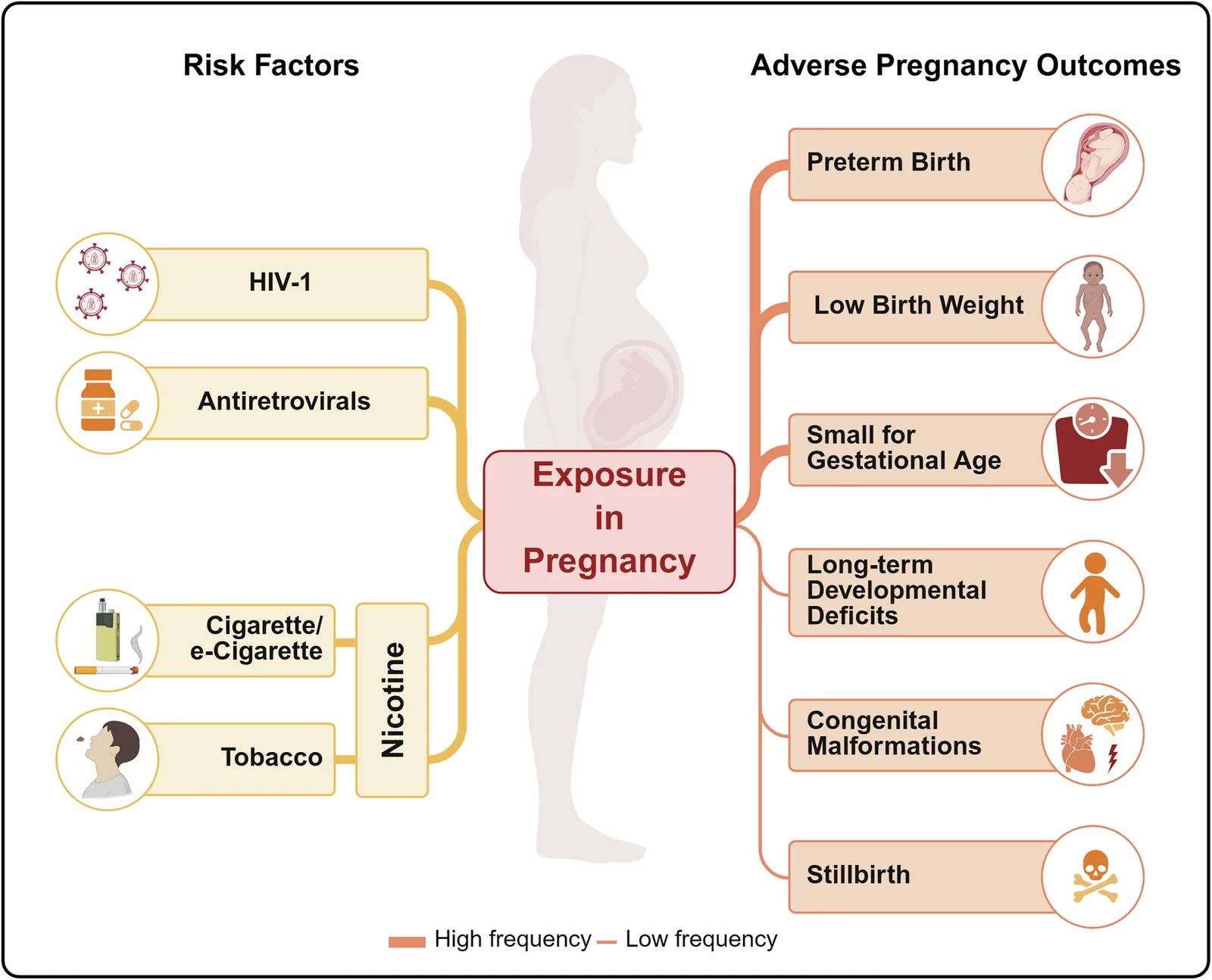
Gestational HIV-1, ART, or nicotine exposure-associated adverse pregnancy outcomes. HIV-1 infection, antiretroviral therapy (ART), or nicotine exposure through cigarette smoking, e-cigarette or other tobacco products usage in pregnancy (left column; yellow color) and their linkage to adverse pregnancy outcomes (right column; orange color) is illustrated. Adverse pregnancy outcomes are divided into two categories. These include low (thin line) and high frequency (thick line) rate of each untoward pregnancy outcome.

Indeed, ART is critical in pregnancy for preventing mother-to-child HIV-1 transmission [1, 2, 6]; yet, concerns remain regarding adverse fetal outcomes linked to gestational ARV exposure. ART-associated risks include, but are not limited to, preterm birth, low birthweight, small for gestational age, stillbirth, and long-term postnatal developmental deficits (Fig. 1) [10, 12–16, 21, 22]. There is still a research gap in understanding underlying mechanisms of ARVs-induced placental impairment leading to adverse developmental outcomes. This problem is further complicated by comorbid substance use during pregnancy. Several addictive agents such as methamphetamine, cocaine, cannabinoids, alcohol, and opioids are commonly used by PWLWH. However, globally, nicotine exposure through smoking or tobacco use remains a major comorbidity [23–25]. The prevalence of tobacco smoking in both men and women living with HIV-1 is 2–3 times higher than that in the HIV-1 negative population in both the United States and the rest of the world. Smoking or tobacco use is an independent risk factor for adverse fetal outcomes, such as preterm delivery, low birth weight, intrauterine growth restriction, or neonatal morbidity (Fig. 1) [25–29]. Although the effects of nicotine alone on pregnancy outcomes have been studied, there is a lack of understanding of how its co-exposure can influence HIV-1 or ARVs-linked effects on placenta.

**Fig. 2:**
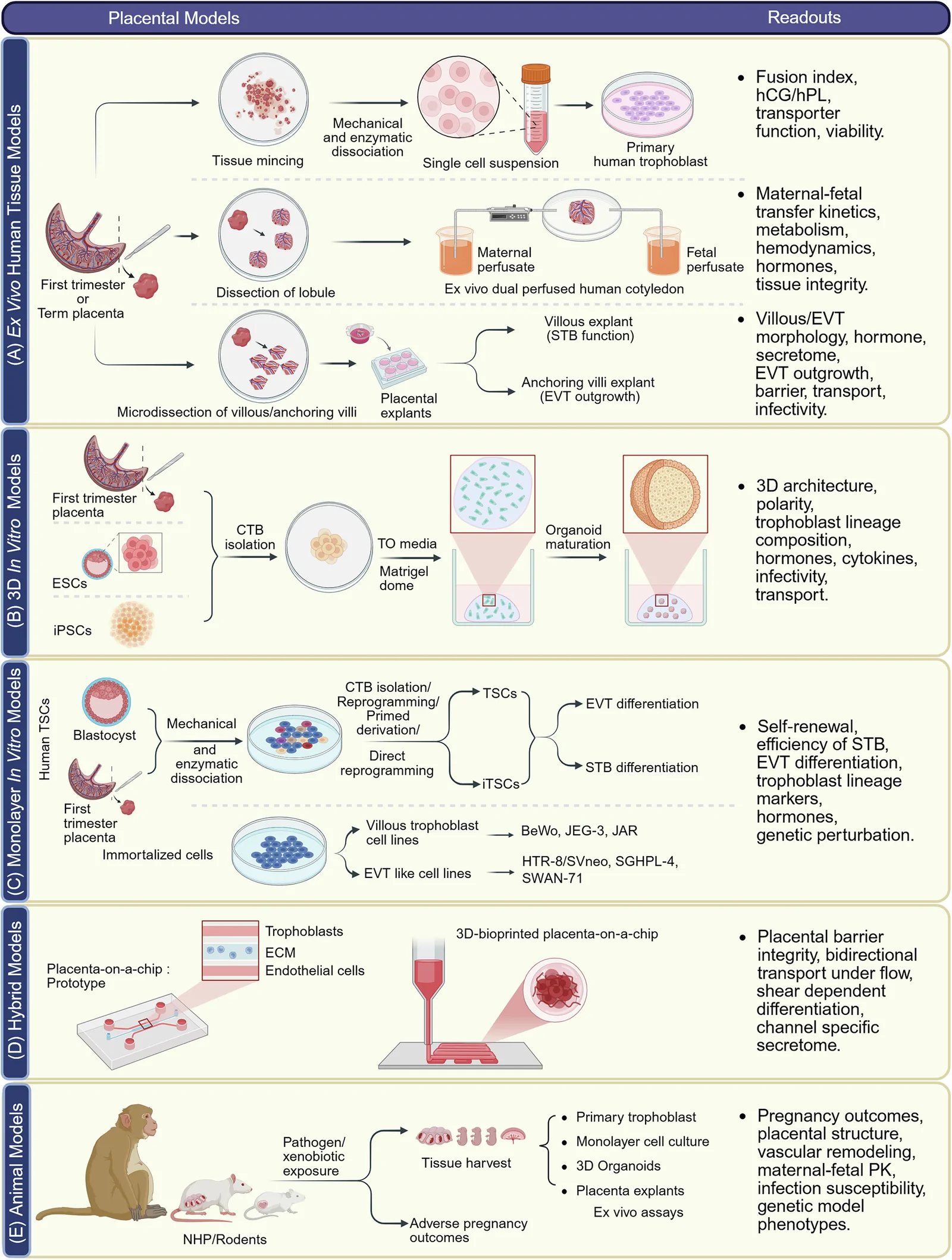
Overview of experimental placental models. Commonly utilized and emerging experimental platforms to investigate placental pathobiology and their respective principal experimental readouts are summarized. **A** Ex vivo human placenta tissue models. These include primary human trophoblasts, ex vivo dual perfused human cotyledon, and placental explants derived from first trimester or term placenta. **B** 3D in vitro models. These include trophoblast organoids (TOs) generated from first trimester placental tissue, embryonic stem cells (ESCs), or induced pluripotent stem cells (iPSCs). **C** Monolayer in vitro models. These include human trophoblast stem cells (hTSCs) derived from blastocyst or first trimester placenta, and immortalized villous trophoblast cell lines such as BeWo, JEG-3, and JAR or extravillous trophoblast (EVT) like cell lines such as HTR-8/SVneo, SGHPL-4, and SWAN-71. **D** Hybrid models. These include placenta-on-a-chip and 3D-bioprinted placenta-on-a-chip systems that incorporate layers of trophoblasts, extracellular matrix (ECM), and endothelial cells under controlled flow. **E** Animal models. These include pregnant rodents (mice and rats) and non-human primates (NHPs) utilized for pathogen or xenobiotic exposure studies. CTB cytotrophoblasts, STB syncytiotrophoblast, hCG human chorionic gonadotropin, hPL human placental lactogen, PK pharmacokinetics, 3D three dimensional.

Appropriate experimental models are essential for uncovering the adverse effects of HIV-1 and associated comorbid conditions on placental physiology, providing mechanistic insights, and developing intervention strategies with translational potential. Currently available placenta models and associated research applications are illustrated in Fig. 2. To date, commonly utilized experimental placenta models have significantly advanced our understanding of cell-specific susceptibility or restriction to HIV-1 infection and the role of inflammatory molecules in regulating HIV-1 replication at the maternal-fetal interface. Additionally, these models have been useful in determining the transplacental transfer of HIV-1 and related proteins, ARVs, and nicotine as well as elucidating potential toxico-pharmacological effects of HIV-1, ART, or nicotine on pregnancy outcomes. However, most of these studies were performed to determine the effects of individual components. Furthermore, relatively new models such as stem cell derived trophoblast organoids and placenta-on-a-chip that emerged superior to commonly utilized models for physiological relevance, are yet to be utilized to study effects of HIV-1 and associated comorbidities. In toto, there is a knowledge gap about whether and how the complex interaction among HIV-1, ART, and nicotine exacerbate the adverse impact on placental development and functions, pregnancy outcomes, and transplacental transmission of each of the HIV-1-ART-nicotine components.

Herein, we provide a comprehensive review of the experimental placenta models that have been or are yet to be utilized to study placental comorbid pathobiology induced by HIV-1 infection, ART treatment, and/or nicotine exposure (Tables 1 and 2). We emphasize how each model is used (or underused) to interrogate infection biology, pharmacokinetic (PK) profile, and toxico-pharmacologic mechanisms. We now share advances made, and remaining challenges through this review to both basic and clinical researchers.

**Table 1:** Overview of placental models.

Model type	Key applications	Readouts	Duration and Throughput	Merits	Limitations	References
Primary human trophoblasts	Fusion and differentiation; endocrine function; innate immunity	Fusion index; hCG/hPL secretion; trophoblast lineage markers; cytokines/EVs	Short term (2–5 days for fused STBs); low–moderate throughput	Preserved genotype/epigenotype; physiologic hormone/fusion behavior	Short lifespan; donor variability; mixed trophoblast lineages; scarce availability of early-gestation tissue	[40, 41, 59, 84–88]
Placental explants (villous/anchoring villi; first trimester)	EVT differentiation and invasion; STB renewal; infection biology; metabolism/transport; inflammatory signaling	hCG/hPL; histology; EV profiling; invasion assays; cytokines	24–72 h static or up to ~1 week with optimized flow; low throughput	Preserved cytoarchitecture and multicellular crosstalk; disease-tissue analysis	Limited longevity, genetic manipulation difficult, and access	[90, 91, 94, 96–98, 100]
Ex vivo dual perfused human cotyledon	Quantitative maternal-fetal transfer (nutrients, drugs, pathogens); placental metabolism; barrier integrity under flow	Bidirectional transfer kinetics; perfusion pressure; leakage/viability checks	≤48 h; very low throughput	Intact villous barrier with separate maternal-fetal circuits; near-physiologic flow	Experimental-technical implementation complexity; finite viability; standardization issues	[118–120, 123, 160]
Immortalized choriocarcinoma cell lines (BeWo, JEG-3, JAR)	Mechanisms of fusion; transporter/TEER studies; molecular signaling; drug effects	cAMP-induced syncytialization (BeWo); hCG; transporter assays; RNA/protein	Long-term; high throughput	Easy, scalable, reproducible	Aneuploid/epigenetically divergent; limited fusion (JEG-3/JAR); lineage ambiguity	[38, 45–47, 49, 50, 52, 90]
Immortalized EVT-like cell lines (HTR-8/SVneo, SGHPL-4, SWAN-71)	Invasion/migration; matrix remodeling; infection/immune function	Invasion through ECM; MMPs; EVT markers; cytokines	Long-term; high throughput	Tractable EVT-like behavior; easy perturbation	Transformed origin; variable lineage relevance	[48, 51]
hTSCs (2D)	Differentiation to STB/EVT; gene function; early placental cell lineage biology	Trophoblast lineage markers; hCG; chromatin state	Weeks-months; moderate throughput	Renewable; stage-tuned differentiation	First trimester bias; requires specialized media; incomplete cellular maturation	[82, 83, 187]
Tissue-derived trophoblast organoids (First trimester vCTB)	Trophoblast lineage specification (CTB/STB/EVT); endocrine function; host-pathogen studies; EV biology	hCG; microvilli; transcriptomic/epigenetic profiling; EVT outgrowth in Matrigel	Months; moderate throughput	Long-term, self-renewing; multicellular trophoblast organization; scalable biobank	“Inside-out” polarity (STB luminal); lacks vasculature/immune cells; First trimester bias	[178, 179, 181]
hTSCs-derived trophoblast organoids	Modeling of trophoblast self-renewal and differentiation; STB and EVT lineage specification; placental development; host-pathogen interactions; drug and toxicant effects	hCG secretion; trophoblast lineage markers; syncytialization; EVT outgrowth/invasion; transcriptomic/epigenetic profiling	Weeks to months; moderate throughput	Renewable and experimentally tractable; derived from purified hTSCs; supports lineage-specific differentiation in 3D microenvironment; cryopreservable	Polarity challenges; immature villous architecture, lack of vasculature, immune cells and late-gestation phenotypes	[43, 81, 82, 191]
PSC-derived trophoblast organoids / (ESC/iPSC)	Placental developmental modeling; genetic relevance; disease variants	CTB/EVT/STB markers; hCG; matrix deposition	Weeks; moderate throughput	Broad availability; isogenic controls; custom genetics	Maturation/polarity challenges; protocol variability	[183]
Placenta-on-a-chip	Transport kinetics across placental barrier	TEER/permeability; shear responses; transporter activity	Days-weeks; moderate throughput	Controlled flow/gradients; modular co-cultures	Limited longevity; dependence on technical expertise	[44,192–198]
Animal models (mouse, rat, NHP)	Placental developmental biology, and hemodynamics; perinatal transmission; PK/PD/toxicity; immune responses	Fetal/placental weights; vascular development; histology; cytokine responses; drug metabolism and transfer kinetics; pregnancy outcomes	Study dependent duration (Flexibility); NHP: low throughput and rodents: high throughput	Integrated systemic physiology	Inter-species differences in placentation and placental development, cost associated with NHPs	[138, 142, 144, 147, 149, 173]

*CTB* cytotrophoblast, *STB* syncytiotrophoblast, *EVT* extravillous trophoblast, *hCG* human chorionic gonadotropin, *hPL* human placental lactogen, *EVs* extracellular vesicles, *TEER* transepithelial electrical resistance, *ECM* extracellular matrix, *MMPs* matrix metalloproteinases, *hTSC* human trophoblast stem cell, *vCTB* villous cytotrophoblast, *PSC* pluripotent stem cell, *ESC* embryonic stem cell, *iPSC* induced pluripotent stem cell, *NHP* nonhuman primate, *PK* pharmacokinetics, *PD* pharmacodynamics.

**Table 2:** Applications of placental models for HIV-1, ART, and/or nicotine exposure studies.

Domain	Model system		Study focus/area	References
HIV-1/ART	BeWo cells		HIV-1 entry mechanisms, transmission, and transcriptional activation	[52, 55, 57, 58, 61, 62]
			ART transfer kinetics and transporter interactions.	[64–68, 70]
	JAR cells		HIV-1 susceptibility and NRTI effects on trophoblast proliferation and endocrine function	[52, 57, 71, 84]
	JEG-3 cells		HIV-1 replication kinetics, LTR activation, and zidovudine-induced cytotoxicity	[52, 57, 71, 84]
	HTR-8/SVneo cells		Effects of ART regimens on trophoblast proliferation and MDR1/MRP1 expression	[68, 73]
	Primary human trophoblasts		HIV-1 co-receptor expression and cytokine-influenced viral transcription	[53, 54, 56, 59, 60, 84]
			Transporter-mediated uptake of abacavir and emtricitabine	[64, 65]
	Placental explants		Characterization of in utero HIV-1 infection and viral localization in trophoblasts and Hofbauer cells	[101, 104, 106]
			ART-induced modulation of placental transporters and folate transport	[108, 109]
			Protease inhibitor (PI) effects on decidualization and spiral artery remodeling	[111]
	Dually perfused human placenta		Maternal-fetal transfer kinetics of ARVs	[126–129, 131]
			Placental barrier integrity against HIV-1 particles and HIV-1p24 antigen	[124, 125]
	NHP	Macaques	Maternal-fetal SIV transmission	[138–140, 142]
			Immune responses and viral load trajectories after fetal SIV exposure	[143]
			Maternal-fetal pharmacokinetics and placental transfer of ARVs	[151–156]
		Patas	Fetal genotoxicity and telomere damage following maternal NRTI therapy	[157]
	Rodents	Mice	EcoHIV pregnancy model for maternal-fetal HIV-1 transmission	[149]
			Postnatal growth impairment and maternal–fetal interactions in HIV-1 proviral transgenic models.	[150]
			ARV-exposure linked developmental toxico-pharmacology	[109, 161–163]
			Imaging biomarkers for ARV-induced embryonic brain metabolomic dysregulation	[164, 165]
		Rats	Zidovudine pharmacokinetics across maternal-placental-fetal compartments	[160]
			BCRP-mediated protection against fetal NRTI accumulation	[158, 159]
Nicotine	BeWo cells		Nicotine-induced oxidative stress, ER stress, and MAPK signaling	[75]
			Nicotine regulation of 11β-HSD2 and lipid transporter expression	[77, 93, 172]
	JAR cells		Carrier-mediated nicotine transport across trophoblast cells	[74]
	JEG-3 cells		Biphasic nicotine effects on trophoblast proliferation	[76]
	HTR-8/SVneo cells		Effects of nicotine and e-cigarette vapor on trophoblast invasion and macrophage polarization	[78–80]
	Placental explants		Nicotine-mediated impairment of trophoblast attachment and outgrowth	[92]
			Smoking-induced ER stress and angiogenic imbalance (sFlt-1/PlGF)	[112, 116, 117]
			Antioxidant protection against nicotine-induced trophoblast dysfunction	[113, 115]
	Human dually perfused placenta		Transplacental nicotine transfer kinetics and metabolism of nicotine to cotinine	[134, 136]
			Nicotine inhibition of amino acid transport and potentiation of PGE2 vasoconstriction	[135, 137]
	Murine embryonic stem cells		Nicotine-induced disruption of placental development via Notch signaling	[35]
	NHPs (Macaques)		Vitamin C mitigation of nicotine-induced placental blood flow defects	[173]
	Rodents	Mice	E-cigarette aerosol effects on uterine blood flow and fetal survival	[166–168]
			JUUL aerosol exposure and impaired lung development in offspring	[169]
		Rats	Nicotine inhibition of trophoblast invasion and placental vascularization	[34]
			Nicotine-induced epigenetic suppression of placental 11β-HSD2	[77]
			Prenatal nicotine exposure mediated disruption of placental cholesterol transport	[172]
			Nicotine-induced ER stress and protein folding impairment	[171]
			Nicotine-mediated abnormal macrophage polarization and trophoblast invasion	[79]
			Hormonal and oxidative stress alterations following e-cigarette exposure	[170]

*ART* antiretroviral therapy, *ARVs* antiretroviral drugs, *CRISPR* clustered regularly interspaced short palindromic repeats, *ER* endoplasmic reticulum, *HIV-1* human Immunodeficiency virus type 1, *LTR* long terminal repeat, *MAPK* mitogen-activated protein kinase, *MDR1* multidrug resistance protein 1, *MRP* multidrug resistance-associated protein, *NHP* non-human primate, *NRTI* nucleoside reverse transcriptase inhibitor, *PBPK* physiologically based pharmacokinetic, *PGE2* prostaglandin E2, *PlGF* placental growth factor, *SIRT1* sirtuin 1, *SIV* simian immunodeficiency virus, *sFlt-1* soluble fms-like tyrosine kinase-1, *11β-HSD2* 11β-hydroxysteroid dehydrogenase type 2, *α7nAChR* alpha-7 nicotinic acetylcholine receptor.

## Trophoblast cell lines

Immortalized trophoblast cell lines are predominantly the first-line in vitro experimental models for studying placental biology, functions, and pathology [30–33]. Ease of propagation and tractability, relative phenotypic stability, and cost-effectiveness make these models well suited for high-throughput studies of trophoblast proliferation, differentiation, invasion, migration, syncytialization, hormone production, and transportation [31, 34]. Choriocarcinoma-derived BeWo, JEG-3, and JAR cells are frequently used as cytotrophoblast-like surrogates, whereas the first-trimester placenta explants-derived HTR-8/SVneo cell line is commonly used as an extravillous trophoblast (EVT) like invasion model [35]. BeWo cells have been utilized for dissecting the molecular control of syncytialization due to their capacity to fuse upon cyclic adenosine monophosphate (cAMP) induction. This functional property makes them essential platforms for evaluating drug transport and metabolism. Conversely, HTR-8/SVneo provides a reliable platform for studying cellular invasion, migration, and signaling pathways in response to pathogen exposures. Additionally, these cell lines have been used to model placental inflammatory signaling and dysfunction relevant to adverse pregnancy outcomes. Studies identified BeWo cells as a source of inflammatory cytokines such as interleukin-6 (IL-6) and interleukin-8 (IL-8), highlighting their relevance as an in vitro model of placental inflammatory responses implicated in pregnancy-related disorders [36, 37]. Moreover, sphingosine-1-phosphate signaling in HTR-8/SVneo cells has been shown to mediate IL-8 upregulation that is required for trophoblast invasion, supporting the use of these cells for investigating molecular mechanisms underlying normal placental function [38]. Taken together, these properties support the use of these cell lines as a relevant, cost- and time-efficient experimental platform for investigating placental molecular and functional responses to pathogen or xenobiotic associated pathology. Thus, herein, we review reports that have utilized BeWo, JEG-3, JAR, or HTR-8/SVneo cells to evaluate the effects of HIV-1, ART, and nicotine.

### HIV-1 infection

Trophoblast cell lines are instrumental in defining placental barrier function. Thus, these models are crucial for elucidating the role of trophoblasts in facilitating or preventing mother-to-child HIV-1 transmission. The susceptibility of trophoblast cells to HIV-1 infection and their contribution to productive viral replication have remained controversial [39–43]. Though cell lines endogenously express CXCR4 and CCR5, they lack or express low levels of cell surface CD4 receptors [39, 44, 45]. Restricted permissiveness to cell-free HIV-1 or limited productive viral replication was noted in BeWo, JAR, and JEG-3 cells [39, 42, 44]. Particularly, in BeWo cells, restriction was observed at the level of cell entry or at early stages of the viral replication cycle [39, 42]. This was evidenced by the instability of internalized viral proteins and the concentration of p24 antigen within endosomal vesicles. However, these cell lines readily supported viral replication following provirus transfection [39, 44, 46]. Furthermore, introduction of inflammatory cytokines or growth factors, including interleukin-1β (IL-1β), tumor necrosis factor-alpha (TNF-α), granulocyte-macrophage colony-stimulating factor (GM-CSF), and epidermal growth factor (EGF) stimulated HIV-1 transcription [39, 47]. In addition, studies evaluating HIV-1 translocation across the trophoblastic barrier using BeWo monolayers have revealed that cell-to-cell contact is often a more efficient virus transmission route than cell-free HIV-1 infection, facilitating the selective passage of viral quasispecies [48, 49]. Altogether, existing literature utilizing cells lines shows that HIV-1 may gain entry into these cells by bypassing the canonical CD4 receptor pathway. This would potentially be through cell-to-cell spread. Nonetheless, productive HIV-1 replication was found to be restricted, highlighting the barrier role of trophoblasts in preventing vertical HIV-1 transmission during gestation.

### ARV effects

To date, only a handful of studies have utilized cell lines to examine the effects of ARVs. Particularly, cell lines have been utilized to determine the transporters involved in uptake of various ARVs, which would potentially facilitate passage of ARVs across placental barrier. In BeWo cells, the uptake of abacavir (ABC) was primarily mediated by the equilibrative nucleoside transporter 1 (ENT1; SLC29A1) [50, 51]; and the uptake of emtricitabine (FTC) was mediated by multiple transporters, including ENT1, concentrative nucleoside transporter 1 (CNT1; SLC28A1), and organic cation/carnitine transporter novel 1 (OCTN1; SLC22A4) [52]. Another study determining transportation of maraviroc (MVC) across the placental barrier utilizing multi-modal experimental system observed cooperative action of efflux transporter, multidrug resistance protein 1 (MDR1), and influx/accumulation transporters including multidrug resistance-associated protein 1 (MRP1), organic anion transporting polypeptide (OATP) 1A2, and 1B3 in BeWo cells [53]. Furthermore, cell lines were utilized to identify ARV interactions with nutrient transporters. These research efforts demonstrated that ritonavir (RTV), saquinavir (SQV), and elvitegravir (EVG) can inhibit sodium (Na^+^)-dependent L-carnitine uptake in BeWo cells [54]; and SQV can increase both the expression and functionality of P-glycoprotein (P-gp) in both HTR-8/SVneo and BeWo cells [55, 56]. An ARV from first-line regimen, bictegravir (BIC), was found to significantly reduce protein levels of System L amino acid transporter isoforms (LAT-1, LAT-2, LAT-4) in syncytialized BeWo cells, leading to impaired L-leucine uptake [57]. While assessing toxic effects associated with ARVs, significant inhibition of proliferation and alterations in hormone production were observed following Zidovudine (AZT) exposure in JAR cells [58]. Others reported AZT-induced mitochondrial toxicity, elevated reactive oxygen species (ROS) levels, and caspase-dependent apoptosis in JEG-3 cell cultures [59]. To investigate potential underlying mechanisms of ART-associated adverse pregnancy outcomes, the effects of individual or combined drug effects of first-line (Atripla; tenofovir (TDF)/FTC/efavirenz (EFV)) or second-line ART (Aluvia; AZT/lamivudine (3TC)/lopinavir (LPV)/RTV) on trophoblast proliferation were evaluated using HTR-8/SVneo [60]. This study confirmed a significant decrease in cell proliferation following exposure to both individual and combined drug components of first- and second-line ART regimen, indicating potential ART-placenta-interaction mediated adverse pregnancy outcomes in women living with HIV-1.

### Nicotine exposure

Trophoblast cell lines have been commonly employed to examine the effects of nicotine and tobacco products. These studies have investigated the effects of nicotine on various cellular functions, including cell survival, proliferation, migration, hormonal signaling, transport pathways, and inflammatory responses, collectively providing mechanistic insights into nicotine induced adverse pregnancy outcomes [61–67]. During early efforts to uncover the mechanism of transplacental nicotine transport, monolayer cell culture of JAR cells was utilized. Nicotine uptake in the JAR cells was found to be saturable, temperature-sensitive, and inhibited by organic cations, such as mecamylamine and quinidine. Overall, this study indicated that the nicotine uptake is a carrier-mediated process [65]. In another study, nicotine was found to induce both oxidative stress and endoplasmic reticulum (ER) stress in BeWo cells, in which nicotine exposure led to a decrease in cell proliferation, increase in the ROS, and elevated the expression of the ER-stress related protein glucose-regulated protein 78 (GRP78). Additionally, nicotine influenced the phosphorylation of mitogen-activated protein kinases (MAPKs), particularly, c-Jun N-terminal kinase (JNK) and p38 [63]. Interestingly, a biphasic effect of nicotine concentrations was reported on the proliferation of trophoblast cells. In contrast to the inhibitory effects on cell proliferation with higher concentrations, a significant increase in the proliferation of JEG-3 cells was observed following exposure to low concentrations (nanomolar) of nicotine and its metabolite, cotinine. Such stimulatory effect could be through activation of extracellular-regulated kinase (ERK) and AKT (protein kinase B) signaling [67]. Moreover, nicotine was found to downregulate placental 11β-hydroxysteroid dehydrogenase type 2 (11β-HSD2) expression by inducing nicotinic acetylcholine receptor (nAChRs)/ERK1/2/ Ets-like protein-1 (Elk-1)/ early growth response-1 (Egr-1) signaling pathway in BeWo cells, providing a potential mechanism of adverse placental development affecting birth outcomes [66].

In addition to elucidating nicotine transplacental transport mechanisms and uncovering adverse effects on placental cellular functions, studies were conducted to assess the effects of electronic cigarettes (e-cigarettes) on EVT functions which are crucial for healthy placenta development. In one of the studies, media conditioned with unflavored e-cigarette vapor significantly reduced HTR-8/SVneo trophoblast invasion and tube formation irrespective of nicotine presence. This study demonstrated cytotoxicity induced by e-cigarette components independent of nicotine, warranting more studies assessing effects of e-cigarette composition on placenta development [62]. Furthermore, in an effort to determine the association between nicotine with preterm labor, researchers employed a co-culture systems utilizing HTR-8/SVneo cells and reported that nicotine regulates abnormal macrophage polarization and trophoblast invasion associated with preterm labor via the α7 nAChR/ sirtuin 1 (SIRT1) axis [64].

Consideration of the comorbid influence of nicotine on the placenta is critical. Periodontitis, a chronic inflammatory condition affecting soft tissues surrounding tooth structure is thought to be associated with adverse pregnancy outcomes. Komine-Aizawa et al. and colleagues examined the comorbid effects of *Porphyromonas gingivalis* lipopolysaccharide and nicotine on invasion function of HTR-8/SVneo cells [61]. The study revealed that *Porphyromonas gingivalis* lipopolysaccharide alone may not have an effect on cell invasion; however, co-exposure of nicotine can exacerbate the inhibitory effect of *Porphyromonas gingivalis* lipopolysaccharide on cell invasion, showcasing the need of studies to evaluate the comorbid effect of nicotine and other infectious pathogens like HIV-1 or medications like ARVs on placental development [61].

While trophoblast cell lines are widely utilized in vitro models for investigating placental physiology and pathology, numerous limitations have been documented that warrant careful consideration during experimental design and data interpretation. These include restricted differentiation, variability in innate immune receptor expression, and genetic divergence from primary tissue. Considering these limitations, for studies investigating underlying mechanistic pathways and screening interventional therapeutic strategies using cell lines, cross-validations using primary trophoblasts, ex vivo placental explants or organoids will be pivotal for accurately delineating independent and comorbid effects of HIV-1, ARVs, and nicotine on placental health.

## Primary human trophoblasts

Primary human trophoblasts are isolated from donated placenta, most commonly from first-trimester or term villous cytotrophoblasts of the chorionic villi [68–70]. These cells have the capacity to give rise to three major trophoblast lineages including cytotrophoblasts (CTBs), syncytiotrophoblast (STB), and EVTs. They show transcriptomes similar to those of corresponding primary trophoblast cells of the placenta. Trophoblasts emerge from the trophectoderm (TE) layer of the blastocyst and subsequently diversify into CTBs, STBs, and EVTs. Briefly, the standard workflow for trophoblast isolation consists of sequential enzymatic dissociation of placental tissue to single cells followed by density-based separation with optional immunoselection to enrich villious CTBs and/or deplete contaminating stromal and immune cells, syncytial fragments, and EVT like populations. These purified CTBs maintain a proliferative, undifferentiated trophoblast phenotype which can be stored for future usage as they retain these phenotypic abilities for many passages or can be plated on extracellular matrix coated substrates (e.g., collagen) in culture medium that supports survival and subsequent differentiation to multinucleated STBs and invasive EVTs. These differentiated lineages can be confirmed by immunostaining or transcriptomic analysis. Commonly utilized trophoblast markers for immunostaining include cytokeratin 7 and tumor protein p63 (TP63) for CTBs, beta subunit of chorionic gonadotropin (CGB) and syndecan-1 (SDC1) for STBs, and human leukocyte antigen-G (HLA-G) and Notch-1 for EVTs [69]. Having transcriptomic and functional similarity to human placenta, primary trophoblasts are commonly utilized to study trophoblast differentiation, hormone production, responses to pathogens, drug transport, and barrier-relevant functions at the maternal-fetal interface [46, 71–77]. Though limited studies to review, these cells possess significant potential to address molecular and functional questions pertinent to HIV-1 infection and its associated comorbidities. In the future, expanded use of primary trophoblast cells is warranted to elucidate the complex interactive impact of HIV-1, ART, and nicotine on each of the trophoblast lineage (CTBs, STBs, and EVTs)-specific physiology. Moreover, primary trophoblasts offer a critical platform for validating results obtained from immortalized cell lines, strengthening both biological relevance and translational potential.

### HIV-1 infection

For improving the understanding of in utero transmission dynamics, primary trophoblast cultures have been employed to determine whether villous trophoblasts support productive HIV-1 infection [46, 71]. A study assessed susceptibility of primary human placental trophoblasts to HIV-1 infection using five different cell-free HIV-1 variants [46]. The cells exhibited resistance to productive HIV-1 infection. This resistance persisted even in the presence of cytomegalovirus coinfection. In contrast, transfection of the same primary trophoblasts with T-cell- and macrophage-tropic provirus plasmids resulted in efficient HIV-1 production. These findings indicated that trophoblast restriction to HIV-1 infection acts predominantly at viral entry or early post-entry stages rather than at downstream replication steps including viral transcription, and assembly.

One more study confirmed these conclusions of restricted HIV-1 replication in primary trophoblasts. To investigate the mechanistic explanation for restricted HIV-1 infection in the placenta, Zachar et al. examined HIV-1 transcriptional competence in primary trophoblasts using long terminal repeat (LTR) activity reporter constructs from distinct HIV-1 isolates [71]. Notably, robust basal and trans-activator of transcription (Tat) induced transcriptional activity was observed [71]. These results demonstrated efficient HIV-1 LTR function in primary trophoblasts, eliminating defective promoter-enhancer activation as the mechanism for the partial restriction to HIV-1 replication observed in these cells. Thus, similar to previous observations in cell lines reported by Kilani et al. [46], this study also indicated potential restriction mechanisms upstream of transcription or at post-transcriptional stages of the viral replication cycle. Overall, the studies utilizing primary trophoblasts cross-validated findings of immortalized cell lines on trophoblast-specific barriers that limit HIV-1 replication at the maternal–fetal interface.

### ART and nicotine exposure

In contrast to studies evaluating HIV-1 replication, no reports were identified that used primary trophoblasts to examine the independent effects of ART or nicotine, or the combined effects of HIV-1, ART, and nicotine co-exposure. Nonetheless, this in vitro model offers several potential high-value applications for addressing critical knowledge gaps related to the individual or combined exposure effects on the placenta. These include (a) defining ARV- or nicotine-driven perturbations in CTB differentiation, syncytial fusion during STB formation, and endocrine function (human chorionic gonadotropin (hCG) and human placental lactogen (hPL) secretion); (b) measuring exposure-induced cytotoxic effects including oxidative stress, mitochondrial dysfunction, ER-stress, and inflammatory signaling; (c) evaluating changes in transporter expression and function as well as trophoblast responses to coinfections in presence of inflammatory cues relevant to HIV-1 infection; and (d) profiling trophoblast-specific extracellular vesicle release and cargo under healthy or diseased conditions as potential biomarkers of adverse fetal outcomes.

Despite having high physiological relevance, primary trophoblast culture remains an in vitro model with several experimental and biological limitations. These include contamination of residual non-trophoblast cells, short-lived culture, and lack of high-integrity monolayers required for barrier assays [72–76]. Potential culture “rescue” interventions for improvements may introduce tradeoffs. For example, efforts to improve cell viability may affect fusion efficiency. Furthermore, donor-to-donor variability and gestational stage dependent differences require careful study designs and interpretation of outcomes [72–76]. Another important challenge arises from tissue accessibility; especially with procurement of early gestation placenta remaining limited and ethically regulated. Findings from the primary trophoblast cultures may be further strengthened by pairing with complementary platforms such as placental explants, and three-dimensional (3D) organoids. These complementary experimental models would not only cross-validate findings from monolayer cultures of primary trophoblasts but improve the physiological relevance of the findings by enabling investigation for prolonged period and determining influence from immunological modulation and multicellular microenvironment.

## Placental explants

Due to preserved 3D architecture and multicellular microenvironment, human placenta explants enable a physiologically relevant investigation of trophoblast differentiation, functions, immune interactions, transport, metabolism, and responses to pathogen or toxin exposure [76, 78–80]. Intact chorionic villous fragments from first-trimester to term human placenta are typically dissected and cultured on supportive substrates, such as collagen I, Matrigel, or gelatin at an air–liquid interface, or on Transwell inserts under physiologic oxygen conditions, with the culture configuration tailored to addressing specific research questions [76, 78, 79]. Placenta explants obtained at early gestation generally utilized to study CTBs renewal and differentiation as well as EVTs generation and their primary invasion function. Whereas explants obtained at later period of gestation or at term are frequently applied to investigate STBs related functions such as hormone secretion (hCG and hPL) and extracellular vesicle release [78, 79, 81, 82]. In addition to direct culturing of intact villous tissue, STB degeneration that occurs ex vivo can be leveraged experimentally. Culturing explants following digestion of the outer layer of STBs can lead to formation of a new layer of STBs within 48 h, making it a suitable model for studying the mechanism of CTBs differentiation to STBs and linked functions [77, 83, 84]. Thus, due to these experimentally advantageous features, placenta explants have been extensively used to probe placental susceptibility and responses to various viruses [85, 86]. Studies included evaluation of viral replication, spread, innate immune activation, apoptosis, and antibody-dependent effects. Due to successful utilization to study viral pathology, placenta explants have also been used to test efficacy of antivirals, vaccines, or hyperimmune preparations [87–89]. Of relevance to the present review, chorionic villous explants have offered a robust ex vivo system to uncover the adverse effects of HIV-1, ART, and nicotine on placental physiology and the cellular and molecular mechanisms that influence these effects.

### HIV-1 and ART

Human chorionic villous explants from first trimester through term have been used to investigate HIV-1 infectivity, effects of inflammatory modulation on viral replication, and viral persistence at the maternal-fetal interface to define the role of the placenta in vertical virus transmission. In earlier studies conducted in the 1990s, multiple research groups evaluated infectivity of free HIV-1 in first trimester and term placenta explants. In contrast to several reports utilizing trophoblast cell lines or primary cells, these studies reported productive HIV-1 infection in both first and third trimester placental explants following exposure to free HIV-1 [90, 91]. However, these studies were limited by a lack of assessment of the underlying mechanisms for viral entry and spread. Recently, a study utilized term chorionic villous explants obtained from women with HIV-1 on prenatal ART to determine the persistence of HIV-1 reservoir and underlying mechanisms for virus spread in the placenta. A latent HIV-1 reservoir was noted in these placenta explants. Upon reactivation with chemical agents in ex vivo culture, the generated mature HIV-1 particles infected non-CD4+ cells in a syncytin-dependent pathway. Overall, this study suggested that viral entry can occur in placenta trophoblasts using a cell-to-cell spread mechanism. Particularly, syncytin that is expressed on placental trophoblasts induces fusion of these cells with HIV-1 infected CD4 + T cells to directly facilitate HIV-1 entry. This innovative work uncovered how HIV-1 overcomes the receptor restriction and enters trophoblasts through cell-to-cell spread mechanism, highlighting the virus transmission and persistence in placenta [92].

Studies also evaluated the influence of inflammatory modulation on HIV-1 infectivity in both first- and term-placenta explants. A study utilizing villous fragments of term placenta reported that TNF-α can enhance viral replication in the placenta after initial restriction for viral entry has been overcome [93]. Another study utilizing first trimester explants also confirmed role of inflammatory cytokines TNF-α, IL-1β, and IL-6 in enhancing HIV-1 replication [94]. Overall, these studies highlighted the critical role of inflammatory signaling for influencing susceptibility to HIV-1 infection at the placental barrier. Furthermore, placental explants have served as an ex vivo platform for determining the cellular susceptibility and restriction to HIV-1 infection in the placenta. A study compared localization of HIV-1 between term placenta infected with HIV-1 in utero and term villous explants from non-HIV-1 pregnancies infected with HIV-1 ex vivo. In situ PCR confirmed localization of HIV-1 DNA in STBs and Hofbauer cells of placenta tissues of both groups, suggesting potential selective virus tropism in the placenta [95]. In another study, using decidual explants, researchers confirmed that the balance of pro- and anti-inflammatory cytokines within the decidua shapes the phenotype of decidua basalis macrophages (dM) and modulates their susceptibility to HIV-1 infection. Particularly, the study showed that the decidual microenvironment including persistence of interferon-γ (IFN-γ) and expression of activated toll-like receptors (TLR)-7 and -8 modulates the low susceptibility of dM to productive HIV-1 infection [96]. Though there is a limited body of literature, studies employing human placenta explants from different stages of gestation have significantly advanced our understanding of cell type-specific susceptibility to HIV-1 infection, tissue resident restriction mechanisms against HIV-1 replication, and inflammatory signaling pathways that enhance virus replication.

In addition to evaluating the susceptibility to HIV-1 infection, placental explants have been instrumental in assessing how ARVs modulate the placental barrier. The effects of ART on placenta transporters were examined using placental villous explants obtained from term pregnancies of healthy women and women with HIV-1, majority on protease inhibitor (PI) based regimen during pregnancy [97]. This study evaluated expression of uptake and efflux transporters on placenta tissues. The researchers observed significant downregulation of adenosine triphosphate (ATP) binding cassette (ABC) transporters (ABCG2, ABCC1,2,4) and solute carrier SLC transporters (SLC21 A9, SLC22A1,3,11) and upregulation of ABCB1 and SLC21A12 transporters. This study highlighted the potential complex interplay of HIV-1 and ARVs on placental transporters, suggesting impairments in transplacental ARV PK and exchange of nutrient and waste products critical for healthy fetal outcomes. Additional studies will be needed to determine the clinical impact of such alterations in expressions of transporters on fetal safety [97]. Due to the potential risk of neural tube defects (NTDs) linked to periconceptional dolutegravir (DTG) usage, significant efforts were employed to evaluate the association between folate and DTG using different study models. This question has been a high priority as DTG is a first-line ARV during pregnancy. Researchers investigated the potential interactions between DTG and folate delivery pathways in the placenta. Particularly, the effects of DTG exposure on the mRNA expression of the reduced folate carrier (RFC), proton-coupled folate transporter (PCFT), and folate receptor-α (FRα) were evaluated on human first trimester placental explants from healthy donors. The clinically relevant DTG exposure was associated with a significant upregulation of RFC and downregulation of FRα. The study indicated that the interaction of DTG with folate transport pathways in the placenta can potentially impact folate delivery to the fetus [98]. However, to date, no definitive cause-and-effect relationship has been identified between DTG and folate. Two studies examined the toxicity linked to AZT, an alternative ARV during pregnancy, using chorionic villous explants from first trimester or term placenta. Studies reported AZT-induced apoptosis and intracellular ROS. Moreover, altered levels of tissue metabolizing enzyme activity were observed. These included increase in the activity of cytochrome P450 1A (CYP1A), β-glucuronidase, CYP reductase (CYPR), and glutathione S-transferase (GST) enzymes but decrease in the activity of uridine diphosphate-glucuronosyltransferase (UGT) enzymes. All of which raise concerns about safety of AZT in pregnancy [59, 99]. As poor pregnancy outcomes have been observed in PWLWH exposed periconceptionally to PI-based ART, recently, the effects of LPV and darunavir (DRV) based ART on human first-trimester placenta-decidua explant co-culture were evaluated at clinically relevant combinations. Interestingly, LPV-based ART was associated with impaired spiral artery remodeling and trophoblast migration. However, no adverse effects were noted following DRV-based ART treatments, indicating its potential safety profile in pregnancy [100].

### Nicotine exposure

Placental explant models have also played a critical role in determining mechanisms underlying toxicity associated with nicotine, cigarette smoke or electronic cigarette (e-cigarette) components exposure at the maternal-fetal interface. Huovinen M. et al. employed first trimester and term human placenta explants to investigate nicotine-linked toxicological effects [101]. Researchers were particularly focused on evaluating effects on ER stress. This study observed a significant increase in GRP78 protein expression in both first trimester and term placenta following nicotine exposure. The study identified a pathway of ER stress through which nicotine can potentially impact placental functions, affecting fetal outcomes [101]. Another research group reported a nicotine-linked decrease in CTB differentiation using first trimester chorionic villous explants obtained from healthy, non-smoking women. Additionally, the same study identified the inhibition of matrix metalloproteinase-9 (MMP-9) as a potential underlying mechanism for nicotine associated inhibition of CTB invasion using isolated purified human CTBs. The study suggested that nicotine exposure during early pregnancy can affect trophoblast pathways critical for EVT generation and uterine invasion [102]. The same research group later evaluated whether nicotine exposure can cause premature depletion of the CTB stem cells utilizing a similar ex vivo experimental platform containing first trimester explants cultured on the Matrigel substrate. Nicotine treatment, at high concentration exposure (6 µM), consistently reduced the expression of various cell cycle markers (cyclin D1, CDK4), mitosis (histone H3), and incorporation of the thymidine analog BrdU with no notable effect on apoptosis [103]. Both studies indicated an adverse impact of nicotine exposure on early pregnancy CTBs proliferation and differentiation, contributing to deficits in placental function and consequently fetal development [102, 103]. Furthermore, a potential therapeutic benefit of vitamin C, vitamin E, and N-acetylcysteine (NAC) to control nicotine-linked oxidative stress was noted in first trimester explants. Such therapeutic effects of vitamins could address nicotine-induced adverse pregnancy outcomes. However, this study was preliminary and warrants further comprehensive evaluations to support the notion [104]. The prevalence of vaping is rising in young adults; however, their safety in pregnancy is unknown. Thus, the effects of e-cigarette constituents (propylene glycol [PG], vegetable glycerin [VG]) alone or in combination with nicotine were evaluated using term placenta explants. The combination of nicotine and PG/VG constituents was found to affect levels of placenta health biomarkers such as IL-6 and β-estradiol (E2), suggesting potential harm of vaping on pregnancy and fetal outcomes [105]. Another study employed term villous explants to examine the impact of cigarette smoke extract on angiogenic markers. Following culture with cigarette smoke extract, conditioned media analysis by ELISA demonstrated a dose-dependent reduction in soluble fms-like tyrosine kinase-1 (sFlt-1) secretion, resulting in a comparatively pro-angiogenic profile with relative placental growth factor (PlGF) abundance. The study claimed that such reduction in soluble vascular endothelial growth factors may explain the observation of reduction in preeclampsia events by cigarette smoking; however, lacked a comprehensive assessment to support the conclusion [106].

To date, studies utilizing human placenta explants investigated HIV-1, ARV, or nicotine exposure as independent perturbations rather than as interactive comorbid insults. HIV-1 focused villous explant studies have defined susceptibility and highlighted inflammatory cytokines as key amplifiers of viral replication. Whereas ARV and nicotine exposed explant studies have primarily evaluated trophoblast differentiation, stress signaling, and transporter regulation under individual exposure conditions. Despite their high physiological and genetic relevance, human placental explants remain underutilized for studying HIV-1 and related comorbidity risks. Consequently, there is a critical knowledge gap in understanding whether and how co-exposure of HIV-1, ARV, and nicotine modulate trophoblast function, barrier integrity, immune signaling, and placental transport/metabolic capacity within the multicellular microenvironment, limiting our ability to predict adverse pregnancy outcomes and develop targeted therapeutic strategies.

Though placenta explants provide a physiologically relevant environment which cannot be fully recapitulated by monolayer cultures of cell lines or primary trophoblasts, some considerations are needed for experimental approaches involving placental explants. The placenta is comprised of a multi-cellular environment which limits cell type-specific genetic manipulation. Also, as trophoblast proliferative capacity declines with gestational age, and ex vivo tissue viability is finite, the choice of placental explants from different gestational ages should be dependent upon the need of the experimental goals. Furthermore, explants cannot be reliably cryopreserved, and access particularly to first trimester tissues remain restricted by ethical constraints. These can affect overall rigor and reproducibility [76, 78, 79]. For the placenta explants, the proposed best practices are to match gestational age and culture configuration according to the biological endpoint, monitor tissue quality over time, and integrate explant findings with complementary model systems to balance physiological fidelity with mechanistic precision [76, 78, 79].

## Ex vivo dual perfused human placental cotyledon

Despite its broad applications, the ex vivo dual-perfused human placental cotyledon model has been primarily employed to quantify transplacental transfer of therapeutic drugs, xenobiotics, and pathogens [107–109]. The preserved structural integrity of the villous tree and maintained independent and functional maternal and fetal circulations enable such investigations. In the dual perfusion approach, both maternal intervillous and fetal capillary networks of an intact term cotyledon are individually cannulated and perfused with peristaltic pumps. The perfusion can be conducted in an open (non-recirculating) or closed (recirculating) configuration [107, 108]. The closed configuration is commonly preferred for studying transplacental transfer of study compounds because it provides controlled conditions and higher sensitivity compared to open configuration. The recirculation of maternal and fetal perfusates allows the precise quantification of transplacental transfer, placental accumulation, and metabolism of the study compound. However, both open and closed circuits have been employed to study transfer of drugs across placenta. One of the key strengths of perfusion-model-derived datasets consists of integration into pregnancy-specific physiologically based pharmacokinetic (PBPK) models, enabling dynamic prediction of fetal PK profiles rather than reliance on static cord blood-to-maternal blood concentration ratios [110, 111]. Moreover, this model enables identification of key determinants associated with transplacental drug transfer, including protein binding, transporter-mediated flux, and placental tissue retention [108, 112]. Collectively, both circuits of the dual-perfused human placental cotyledon model allow clinically relevant prediction of placental drug retention and fetal drug exposure, aiding to understand drug safety in pregnancy. To date, researchers have taken advantage of this unique physiologically relevant ex vivo system to study transfer of cell-free HIV-1 particles, viral antigens, ARVs, or nicotine across placental barriers.

### HIV-1 particles and ARVs

As discussed earlier, molecular mechanisms of trophoblast HIV-1 infection can be investigated using cultured immortalized trophoblast cell lines, primary human trophoblast cells or human placental explants. While these models can be utilized to study placental barrier properties, ex vivo dual-sided placental perfusion is a far more superior and physiologically relevant experimental platform for studying the mechanisms of HIV-1 transfer from mother to fetus during gestation. The first study by Bawdon et al. evaluated the transplacental transfer of cell-free HIV-1 and HIV-1 p24 antigen using perfusion model [113]. Nine placenta samples obtained from uncomplicated pregnancies at term by vaginal or cesarean section deliveries were employed. Infectious cell-free HIV-1 was not detected in any of the fetal perfusate samples. Interestingly, low concentrations of HIV-1 p24 antigen were detected in fetal perfusate samples from three out of nine placentas. The same group further investigated placental passage of HIV-1 p24 antigen to the fetal circulation using a similar ex vivo placenta perfusion model [114]. The study observed transfer of virus antigen from maternal to fetal perfusate in a concentration dependent manner. Both studies together indicated that transplacental passage of intact cell-free HIV-1 particles is restricted; however, transfer of viral p24 antigen across placenta may be possible. Studies also suggested that the presence of p24 antigen in fetal circulation may not parallel with HIV-1 infection [113, 114].

In contrast to placental HIV-1 transfer evaluations, the dual-perfused human placental cotyledon model has been widely utilized to investigate transfer of ARVs across placenta. These research efforts have provided clinically relevant information on potential fetal ARV exposure and subsequent drug safety, and pre-exposure prophylaxis activity. The model has been employed to study transplacental transfer of ARVs from several classes including integrase strand transfer inhibitors (INSTIs), PIs, nucleoside/nucleotide reverse transcriptase inhibitors (NRTIs), non-nucleoside reverse transcriptase inhibitors (NNRTIs), entry inhibitors (CCR5 antagonists), fusion inhibitors, and PK enhancers. Comprehensive details of all ARVs evaluated using ex vivo placental perfusion models are reviewed elsewhere [108]. Herein, we discuss studies focused only on drugs from the INSTI class as this class consists of relatively newly approved ARVs and the majority of them are currently recommended during pregnancy such as BIC, DTG, and raltegravir (RAL). This focused approach will allow us to efficiently articulate the significance of the placenta perfusion model in HIV-1 research while avoiding any redundancy of information reviewed by others. Two studies utilized a closed-circuit configuration of the ex vivo human dually perfused cotyledon model to determine transplacental DTG transfer. Both studies confirmed moderate to high DTG transfer from mother to fetus with a mean fetal-to-maternal ratio of 34 and 60%, warranting further investigations of potential fetal toxicity associated with in utero drug exposure. [115, 116]. Like DTG, placental transfer of RAL was found to be high in an open circuit perfusion model with a fetal transfer rate (FTR) of 9.1 ± 1.4% (mean ± SD) [117]. Another drug from the INSTI class, EVG was found to cross the placenta at a moderate level. A study utilizing the dually perfused cotyledon model with both open and closed circuit reported placental transfer of EVG with FTR of 19 ± 13% and 20 ± 10% (mean ± SD), respectively. Additionally, EVG accumulation was noted in the placenta tissue, raising the concerns of potential toxic effects on fetal and placental metabolic health [118]. Clinical reports have affirmed the high transplacental transfer of DTG, RAL and EVG, highlighting the application of the ex vivo dual-perfused human placental cotyledon model in predicting clinically relevant ARV kinetics at the maternal-fetal interface [119]. In contrast to other INSTIs, low transplacental transfer rate of BIC and cabotegravir (CAB) in an open-circuit perfusion model was reported [120]. Median maternal-to-fetal ratios of 7% (6–9.5%) and 10% (5–16%) were observed for BIC and CAB, respectively. This study highlighted potential safety benefits associated with reduced fetal drug exposure and, simultaneously, noted possible limitations with reduced pre-exposure prophylaxis efficacy in fetuses due to restricted transplacental transfer. Interestingly, low transfer of BIC using the placenta perfusion model contrasted with high transplacental transfer of BIC observed in a human case report [121]. Long-acting CAB injectable is currently not recommended in pregnancy, due to lack of data on safety, dosing, and PK during pregnancy. However, its usage in pregnant women at a large scale is expected in the near future due to known high efficacy properties in adults, especially in cisgender women [122]. Therefore, more investigations are needed to understand the transplacental transfer, safety and pre-exposure prophylaxis benefits of BIC and CAB in pregnancy.

### Nicotine

The ex vivo human cotyledon perfusion model has been utilized to quantify transplacental permeability of nicotine, retention, and bioconversion of nicotine to its major metabolite, cotinine, for determining potential mechanisms of smoking linked adverse pregnancy outcomes [123–126]. To determine placental nicotine permeability, one of the earlier studies evaluated transplacental transfer and metabolism of nicotine using both open and closed-circuit configurations of the dually perfused human cotyledon model [125]. The study detected nicotine in the fetal circulation within 5 min following introduction of nicotine into the maternal circulation. The study reported that nicotine not only readily crosses the placenta but also accumulates in placental tissue at a higher degree. At the end of 2 h-perfusion in closed circuit configuration, the total nicotine recovery was 48.7 ± 6%, 20.6 ± 4.8%, and 30.7 ± 6.1% from maternal circulation, fetal circulation, and placental tissue, respectively. Furthermore, there was no evidence of placental metabolism of nicotine to cotinine. Altogether, this study suggested that adverse fetal outcomes may be due to direct exposure to nicotine [125]. Another study by Sastry et al. and colleagues also observed consistent results with nicotine transfer using closed-circuit configuration of perfusion model [123]. The study noted rapid placental transfer of nicotine. 18.6% of the nicotine added to the maternal perfusate was transferred to the fetal perfusate within 80 min of perfusion. Also, less than 1% of nicotine metabolism to cotinine was measured [123]. Later, same group examined whether influence of nicotine and cotinine on prostaglandin E2 (PGE2) affects placental vasculature and the activation of placental phospholipase A2 (PLA2). PGE2 is a key regulator of placental vascular function and thus is known to induce labor and initiate uterine contractions. Using the perfused human cotyledon platform, researchers observed that cotinine was more potent than nicotine in potentiating the vasoconstrictive effects of PGE2 on fetal placental circulation and activating PLA2. Together, the study proposed that cotinine accumulation in fetal circulation may contribute to production of PGE2, causing induction of preterm labor and spontaneous abortions [124]. In addition to determining placental nicotine passage, the effects of nicotine on placental amino acid transport were assessed as an underlying mechanism of intrauterine growth restriction using closed-circuit placental perfusion model. Nicotine significantly inhibited the transport of arginine. The study also evaluated the combination effects of nicotine and cocaine. The inhibitory effect of combination treatment was observed on alanine, arginine, phenylalanine, and valine transport. Researchers concluded that both nicotine and cocaine, alone or in combination, may affect placental amino acids transporters that are necessary to maintain the nutrient gradients essential for normal fetal growth [126].

Even with high clinical relevance, the cotyledon perfusion model is largely limited to term placentas. The viable experimental window is finite, and the study outcomes can vary due to inter-placenta heterogeneity and diversity in implemented methodologies. Transplacental transfer measurements are sensitive to experimental conditions. Protein binding, flow rates, circuit configuration, and the sampling duration can each influence the apparent transfer of a study compound. In general, measurements of drug transfer using ex vivo perfusion model corresponded well to in vivo findings; yet further standardization of the technique would improve reproducibility and facilitate an effective prediction of drug PK in pregnancy.

## Animal models

While in vitro cell cultures, and ex vivo tissue explants offer valuable mechanistic insight, animal models remain necessary to study integrated systemic physiology and investigate pathobiological responses at the maternal-fetal interface. In addition to capturing influences of HIV-1, ART, or nicotine at the maternal-fetal interface, animal models have allowed longitudinal evaluation of pathology progression, drug PK profile, immune responses, transplacental transfer, and developmental (prenatal and postnatal) outcomes [127–130]. Non-human primate (NHP) and rodent models including mice and rats have been utilized to study these complex interactions for defining maternal-fetal health, investigating the role of placental barriers, and screening novel therapeutic means during pregnancy [98, 129, 131, 132].

### HIV-1 infection

Due to the lack of robust rodent models to study HIV-1 infection dynamics in pregnancy, to date, NHP models have been proved to be very critical in assessing experimental evidence that lentiviruses can cross the placenta, though in a restricted manner, and establish fetal infection prior to birth [133]. NHP models have enabled rigorous assessments of not only prenatal (transplacental), but also intrapartum (at delivery) and postnatal (through breastmilk) simian immunodeficiency virus (SIV) transmission routes [134, 135]. Foundational studies discussed below demonstrated that maternal SIV infection can lead to in utero fetal infection, revealed selective viral passage during vertical transmission, and established the placenta as a biologically restrictive but permeable barrier to lentiviral spread.

Ochs et al. carried out an early proof-of-concept study using three pregnant pigtailed macaques (*Macaca nemestrina*) model to investigate whether SIV transmits from the mother to the fetus in utero [136]. Following virus inoculation during third trimester, SIV infection was detected in one of three infants shortly after birth. The study provided evidence that SIV can cross the placenta in a restricted manner and establish infection in the fetus in utero. Furthermore, Amedee et al. utilized five pregnant female macaques to examine viral and host factors that contribute to transplacental transmission of HIV-1 [134]. Researchers utilized three species of pregnant macaques (*Macaca mulatta, Macaca fascicularis*, and *Macaca nemestrina*) inoculated with SIV strains during mid-gestation (2nd or 3rd trimester). Pregnant macaques were inoculated intravenously (IV) either with SIV/DeltaB670 or a combination of SIV/DeltaB670 and SIV/17E-Fr. The study further performed the genetic analysis of paired isolates from five SIV-infected pregnant macaques and their in utero-infected infants. Genetically diverse virus populations were noted in all pregnant macaques at parturition, whereas a single genotype from the maternal quasispecies was identified in the infants at birth, indicating selective passage for viral variants through placental barriers. Interestingly, the transmitted genotypes showed macrophage-tropic properties in subsequent in vitro analysis. Overall, the study suggested genotypic and phenotypic selection in transplacental transmission of SIV and potentially a critical role of macrophages in establishing fetal infection in utero. Later, the same group compared these fetal viral genotype data from transplacental infection with viral genotypes in two newborn macaques infected orally with SIV/DeltaB670 and in one infant infected via amniotic inoculation in late gestation [137]. Researchers utilized pigtailed macaques (*Macaca nemestrina*) for the comparative assessment, enabling assessment of infection route-dependent viral dynamics [137]. Interestingly, in oral or amniotic infected models, more than one viral genotype was observed in each infant, suggesting different mechanisms in establishment of viral infection for these routes compared to selective passage to viral variants during establishment of transplacental fetal infection. In contrast to these studies, other studies using pregnant rhesus macaques (*Macaca mulatta*) suggested that the key transmission routes of SIV infection from mother to its fetus are potentially at parturition or through breastfeeding rather than in utero [133, 138]. Recently, a study examined whether in utero fetal exposure to infectious pathogen can result in reduced immune responses or tolerance to those pathogens when exposed postnatally [135]. During the first trimester, fetal rhesus macaques (*Macaca mulatta*) were injected under ultrasound guidance using an intraperitoneal (IP) approach with nonpathogenic SIVmac1A11 in utero and then challenged with pathogenic SIVmac239 one year after birth. Significantly reduced plasma RNA viral loads were noted in SIVmac1A11-injected animals compared to the control group up to 35 weeks post-infection. Additionally, animals exposed to SIVmac1A11 in utero had lower levels of PD-1 on CD8 + T-cells, and more circulating Th17 cells in comparison to controls. Altogether, the study did not observe the development of tolerance following in utero exposure to pathogen. However, distinct immune responses controlling viral replication following postnatal SIV challenge were identified.

In general, rodent models enable high-throughput screening under controlled experimental conditions for improving mechanistic insights into viral progression and pathology. However, currently the research field lacks a reliable rodent model that recapitulates HIV-1 replication, placental transmission, and pregnancy-specific maternal–fetal pathobiology [132]. This absence of a translationally relevant rodent model to study HIV-1 pathobiology in pregnancy has created a significant barrier to mechanistic and therapeutic investigations. In general, normal mice strains utilized in preclinical research do not get infected with HIV-1 virus. Thus, for past two decades, different humanized mice models reconstituted with human immune cells have been widely utilized to study cell-free HIV-1 infection linked pathology, virus reservoirs, treatment, prevention, and cure strategies [128, 130, 139–141]. Unfortunately, these humanized mice cannot breed as irradiation modulation is one of the critical steps in the generation of these models. Thus, humanized mice cannot be used for pregnancy related research. More efforts are critically needed to improve the humanized mouse model generation process which would allow their utilization for pregnancy research. However, to date, immunocompetent mice infected with the EcoHIV or transgenic mice expressing non-infectious HIV-1 provirus have been utilized to address some aspects of HIV-1 infection associated pathobiology in pregnancy [132, 142].

Recently, Ranjbar et al. and colleagues introduced a new rodent pregnancy model for HIV-1 research, in which female C57BL/6 J mice were infected with EcoHIV, a chimeric ecotropic HIV virus [132]. Mice were injected with 2.5 × 10^6 ^pg/mL of EcoHIV virus either IP around one week before mating or IV during pregnancy at gestation day (GD) 11.5. EcoHIV infection was detected in 90% of pregnant mice inoculated prior to pregnancy and 100% of pregnant mice inoculated during pregnancy. EcoHIV infection was associated with reduced maternal weight gain, lower fetal weight, higher placenta weight, and lower placenta efficiency. Importantly, perinatal transmission of EcoHIV from mother-fetus was limited. Transmission rate was 3.1% following virus inoculation during pregnancy and 17.9% following virus inoculation prior to mating. This model showed potential for studying HIV infection-linked adverse effects on maternal, placental, and fetal health outcomes. Another research group employed HIV-1 transgenic mice expressing replication-defective proviral genome to investigate how the maternal-fetal environment modulates HIV-1–associated developmental outcomes during pregnancy [142]. Transgenic mice had gag/pol deletion mutant of HIV-1 pNL4-3 provirus. Mice homozygous for the HIV-1 transgene had normal intrauterine and birth weights but displayed severe postnatal growth restriction. Transgene expression was prominent in transgenic fetuses, placentas, and neonates. This expression was also associated with reduced postfertilization pregnancy rates in transgenic dams. Altogether, this study showed that the expression of the HIV-1 genome and associated adverse effects on pregnancy or developmental outcomes are potentially influenced by maternal-fetal interactions [142].

### ART exposure

An optimal ARV PK profile is critical for preventing mother-to-child HIV-1 transmission as well as for improving maternal health. However, utilization of ARVs during pregnancy requires careful evaluation because pregnant women are underrepresented in clinical trials, resulting in knowledge gaps regarding optimal PK profiles and drug safety during pregnancy [119]. Physiological changes occurring during pregnancy such as induction of metabolizing enzymes or increase in drug transporter activity leading to drug efflux can drastically reduce maternal drug levels below the therapeutic need [119]. Such alterations in ARVs PK profile can lead to virologic failure. In addition to adequate ARV levels in mother to control virus replication and subsequent prevention of vertical HIV-1 transmission, sufficient transplacental drug transfer in fetus is potentially needed for pre-exposure prophylaxis. However, direct exposure of the placenta or fetus to ARVs in utero may lead to drug-related adverse events and developmental malformations. Thus, animal models play a critical role in preclinical assessments, enabling the investigation of the integrated maternal-placental-fetal PK profile of drugs and their association with drug efficacy and safety during pregnancy.

A pilot study performed in pregnant rhesus macaques was a good example that showed how a drug profile during pregnancy can be a limitation even after its pharmacology is well established in adults [129]. In this study, researchers evaluated whether MVC, a CCR5 receptor antagonist, can effectively cross the placenta and have a long half-life in neonates to serve as a potential alternative candidate to nevirapine (NVP) in a single-dose intrapartum strategy for the prevention of mother-to-child HIV-1 transmission [129]. Both infant and maternal plasma MVC concentrations and MVC-CCR5 receptor occupancy on CD4 + T-cells were determined following administration of an oral dose of MVC to pregnant rhesus macaques 2 h before delivery. The study reported poor transplacental transfer of the MVC with the median fetal-to-maternal area under the concentration-time curve (AUC) ratio of 0.009. Subsequently, a clearance of MVC from the fetal blood was noted within merely 3.5 h. Extremely low expression of CCR5 receptors on CD4 + T-cells of newborn macaque prevented determination of MVC receptor occupancy in infants. Interestingly, in mothers, only partial protection of CCR5 receptors was observed over 24–48 h after dosing. Around 35% of CCR5 receptors no longer had bound MVC. The study suggested that due to poor transplacental transfer and short PK profile in newborn rhesus macaques, a single intrapartum dose of MVC to mother may not be effective in reducing the risk of vertical HIV-1 transmission. Another group investigated whether TDF reaching fetus during gestation causes bone-related toxicity [143]. In this study, pregnant rhesus macaques (*Macaca mulatta*) were administered daily with subcutaneous (SC) chronic dosage of TDF (30 mg/kg). The chronic TDF exposure was associated with reduced fetal body weights and crown-to-rump lengths, and reduction in fetal bone porosity, suggesting that TDF can readily cross placental barriers and may affect fetal developmental outcomes. In addition to rhesus macaques (*Macaca mulatta*), pigtailed macaques (*Macaca nemestrina*) have been employed to determine transplacental transfer of ARVs. PK investigations of AZT, and zalcitabine (ddC) confirmed both ARVs readily cross transplacental barrier [144–146]. Studies estimated that average fetal exposure would be around 75 and 60% of the maternal exposure for AZT and ddC, respectively [144, 145]. Pigtailed macaques were also utilized to determine drug-drug interactions affecting transplacental transfer for potential combination therapies in pregnancy. A study evaluated whether AZT affects the transfer of didanosine (ddI) across placental barriers using near-term pregnant pigtailed macaques. The study did not find any effect of concurrent AZT administration on transplacental transfer kinetics of ddI [147]. Furthermore, the patas monkey model (Erythrocebus patas) has been used for studying adverse effects of ARVs on fetus. Following gestational exposure to combination of AZT and 3TC at human-equivalent dosage, the total DNA damage observed in fetuses of patas monkey exposed to both drugs was at least double to that observed in fetuses exposed to AZT alone. Researchers concluded these findings by noting higher level of drug-DNA incorporation and telomere shortening in fetus organs exposed in utero to combination of AZT and 3TC compared to AZT alone [148].

In rodent animal models, both rats and mice have been commonly used by researchers to determine ARV PK and associated safety profiles in pregnancy. A study utilized pregnant Sprague-Dawley rats to study effect of chronic in utero exposure to AZT or 3TC on expression of MDR transporters including P-gp, BCRP, and MRP2 in placenta and fetal liver, and the subsequent protective role of these transporters [149]. An increase in BCRP expression was observed in both the placenta and fetal liver following chronic in utero exposure to AZT, but not to 3TC. This induction of BCRP expression following AZT exposure paralleled with reduced AZT accumulation in fetal tissue and reduced mitochondrial toxicity and DNA damage. In contrast, no effect of chronic 3TC exposure on any of the MDR transporters resulted in accumulation of high 3TC levels in fetal liver cells and DNA damage, signifying the protective role of MDR transporters, particularly BCRP, for AZT in pregnancy [149]. The same research group also examined the effect of in utero AZT exposure on ABCG2 expression and its protective role utilizing same chronic exposure model in Sprague-Dawley rats [150]. Researchers observed increase in ABCG2 expression in both placenta and fetal brain tissues. Higher expression of ABCG2 was also paralleled with decrease in AZT accumulation and protection of mitochondrial function in fetal brain. Both studies, together, underscored the critical role of drug transporters in regulating drug accumulation in placenta or fetal tissues and in mediating protective functions. In addition to determining the effects of transporter proteins, rat models have been utilized to determine ARV PK profile at maternal-fetal interface. In earlier days, pregnant Long-Evans rats were employed to investigate potential of AZT in crossing placental barriers [151]. The study observed that AZT after oral administration at 30 mg/kg dosage readily crosses placenta with the drug concentration reaching 75 and 58% in the placenta and fetus, respectively, of the maternal serum AZT levels by 2 h after administration. Continued assessment showed that AZT concentration in placenta and fetal liver exceeded maternal AZT levels by 4–6 h following administration.

To date, different strains of mice have been utilized to evaluate the transplacental transfer of ARVs and the consequent effects on developmental outcomes. Sarkar et al. evaluated the effects of in utero exposure to PI-based ART on neurodevelopmental outcomes using pregnant C57BL/6 J mice [152]. Effects of atazanavir (ATV)/RTV/ABC/3TC or ATV/RTV/TDF/FTC on pups’ neurodevelopment were examined following administration to pregnant mice during entire gestation period. Researchers observed that in utero exposure to PI-based ART delays the appearance of primitive reflexes that involve vestibular and sensory-motor pathways in mouse pups during postnatal development. The study suggested that in utero ART exposure could alter the gestational environment, potentially disturbing the maturation of the neurocircuits required for healthy brain development. The same research group also investigated comparative adverse impacts of commonly utilized dual-NRTI backbones including AZT/3TC, ABC/3TC, and TDF/FTC, on placental mitochondria function and oxidative stress using pregnant C57BL/6 J model [153]. Following daily oral administration up to GD 15, AZT/3TC and ABC/3TC were associated with lower fetal and placental weights. Additionally, comparatively, AZT/3TC treatment led to elevation in malondialdehyde levels and decrease in mRNA of manganese superoxide dismutase and peroxisome-proliferator-activated receptor gamma coactivator 1-alpha in the placenta, signifying AZT/3TC induced placental oxidative stress. Interestingly, TDF/FTC backbone combination was noted to be safer in comparison to AZT/3TC for usage in pregnancy. Our own work evaluated PK profiles of DTG during pregnancy using pregnant C3H/HeJ mouse model and measured biodistribution of DTG to placenta and embryo brain tissues [154]. Following daily oral administration of supratherapeutic dosage of DTG (50 mg/kg; mouse weight), average DTG concentrations detected in maternal plasma and paired placenta tissues were 27,296.3 ± 2701.15 ng/mL and 3528.04 ± 316.56 ng/g, respectively, at GD 16.5. The ratio of placental DTG levels to mother’s plasma DTG levels was 0.12. In addition, high DTG concentrations in embryonic brain tissues (average of 663.6 ± 75.13 ng/g) were measured at GD 16.5, and the ratio of embryo brain DTG levels to mother’s plasma DTG levels was 0.024. Our study not only validated clinical reports of high transplacental transfer of DTG but also identified, for the first time, DTG levels in the embryo brain during pregnancy, indicating direct exposure of the embryo brains to DTG during a critical period of development. Notably, we also observed that DTG inhibits matrix metalloproteinases (MMPs) activities in embryo brain during gestational exposure, potentially leading to neuroinflammation and neuronal injury in the brains of mice pups during postnatal development. Overall, the study identified DTG inhibition of MMPs activities as a developmental neurotoxicity biomarker. In addition to identifying potential underlying mechanisms for in utero DTG exposure-linked neurodevelopmental impairments, we evaluated safety and benefits of injectable long-acting (LA) nanoformulations of DTG (NDTG) against daily oral native DTG administration in pregnant C3H/HeJ mice [155, 156]. The study demonstrated that NDTG achieves sustained therapeutic DTG levels in mother’s blood while lowering drug exposure to the placenta and embryo brain. Lowered DTG exposure to fetal brain following NDTG injection in comparison to daily oral DTG administration was further associated with reductions in DTG-linked oxidative stress and amelioration of neuronal and synaptic impairments. Collectively, our work showed that injectable LA formulations would minimize embryo brain DTG exposures due to the associated pharmacological properties, potentially improving drug safety in pregnancy. Our studies highlight the strengths of rodent models in investigating drug-linked toxicity in pregnancy, uncovering underlying mechanisms, and screening therapeutic means to improve safety profiles or develop intervention strategies during pregnancy. Due to the potential association of DTG with developmental neurologic abnormalities in both clinical and preclinical studies, another group also investigated the effect of DTG exposure on the functional expression of the RFC, PCFT, and FRα in pregnant C57BL/6 J mice [98]. The study found that that DTG was associated with an increase in placental RFC and PCFT mRNA expression, accompanied by a decrease in placental FRα mRNA under folate-deficient dietary conditions. These data demonstrated a potential interaction between DTG and folate transport pathways in the placenta, potentially impacting folate delivery to the fetus under folate deficient conditions. However, as mentioned earlier, definitive cause-and-effect relationship between DTG and folate is yet to be established.

### Nicotine/smoking exposure

Due to preserved complex interactions among maternal cardiovascular adaptations, uteroplacental blood flow, endocrine signaling, and fetal development, animal models remain a valuable means to study the effects of nicotine and smoking exposure during pregnancy [127, 131, 157, 158]. With recent advances in experimental procedures, animal models also can be employed to uncover effects of cigarette smoke components and e-cigarette aerosols [157, 158]. Collectively, in vivo studies have been used to define how nicotine and smoking exposure during pregnancy can affect placental structure and function, compromise fetal growth, and potentially impair postnatal cardiopulmonary and neurobehavioral outcomes [127, 131, 159].

Rodent models provide scalable platforms for testing dose, timing, and route of exposure-dependent nicotine effects. Thus, these models have been commonly used by researchers to evaluate placental deficits induced by nicotine, cigarette smoke, and e-cigarette aerosols. The usage of e-cigarettes is on the rise [159, 160]. Around 15% of pregnant women use e-cigarettes containing nicotine and flavoring as a safer alternative to traditional tobacco cigarettes. However, their safety during pregnancy and particularly their effects on placenta remain unknown. A study utilizing pregnant FVB/J mice reported that exposure to sub-ohm e-cigarette aerosols with or without nicotine resulted in maternal diastolic dysfunction, reduced uterine artery blood flow, fetal bradycardia, structural alterations in the placenta, and reduced placental sufficiency [159]. Another group evaluated the effects of flavored e-cigarette aerosols with and without nicotine on the placenta of pregnant C57BL/6 J mice [160]. This study reported that flavored e-cigarette without nicotine increased early fetal demise potentially due to increased blood cell accumulation at implantation sites. Whereas e-cigarette aerosols containing nicotine primarily reduced embryo to placenta weight ratios. Furthermore, in both groups, e-cigarette aerosols with and without nicotine exposure induced varying downregulation of genes critical for placental development, and the degree of impact on these genes was fetal sex dependent [160]. Together, these studies suggest adverse influence of both e-cigarette vehicle components alone and nicotine on pregnancy outcomes. With an increase in usage of e-cigarettes, there is a need for additional investigations to uncover e-cigarette-related placenta toxicity and causal underlying mechanisms. Furthermore, Beck et al. evaluated the effects of secondhand smoke (SHS) and e-cigarette exposure on placental apoptotic and growth-regulatory pathways in pregnant C57BL/6 mice [157]. SHS and e-cigarette exposure created a pro-apoptotic environment in the placenta. An increase in pro-apoptotic markers including BAD (Bcl-2-associated protein) and Fas (CD95)/FasL and a decrease in anti-apoptotic markers such as B-cell lymphoma 2 (BCL-2), survivin, and heat shock protein 27 (HSP27) was determined in placenta tissue, indicating potential pathways underlying impaired fetal development following SHS or e-cigarette exposure. Researchers also have evaluated the effects of nicotine exposure during pregnancy on postnatal organ development. Offspring born to pregnant BALB/c mice exposed to mint flavored JUUL aerosols throughout gestation exhibited impaired lung development and later life susceptibility to airway disorders. Although this study was not solely focused on placenta health and did not indicate a direct association between placenta impairments and lung developmental deficits, it did demonstrate induction of hypoxia and oxidative stress in the placenta, highlighting the effect of JUUL exposure during pregnancy on the gestational environment and its potential association with fetal organ development [158].

Studies utilizing pregnant Sprague-Dawley rats also evaluated effects of e-cigarette aerosol exposure during pregnancy on endocrine and oxidative pathways that may affect fetal development. Maternal e-cigarette inhalation reduced circulating prolactin and increased placental xanthine oxidase activity, with reduced fetal and placental mass [161]. This proof-of-concept study indicated two potential pathways influenced by e-cigarette aerosol which can negatively impact fetal developmental outcome. These included redox imbalance within the placenta tissue and maternal hormonal imbalance [161]. Another study utilized pregnant Wistar rats to test the hypothesis that maternal nicotine exposure leads to both placental ER stress and impaired disulfide bond formation [162]. In this study, rats were exposed to nicotine periconceptionally and SC administration was completed daily. Assessments of rat placenta protein and mRNA levels confirmed augmented ER stress, hypoxia, and impaired disulfide bond formation pathway. This study provided a novel insight into the mechanisms of nicotine exposure-induced placental deficits during pregnancy [162]. Rat models have also been utilized to study effects of nicotine exposure during pregnancy on metabolic and immune pathways that may connect nicotine exposure to adverse birth outcomes. A study by Zhang et al. and colleagues utilized pregnant Wistar rats to elucidate the role of placenta in determining cholesterol levels in prenatal nicotine exposure-induced intrauterine growth retardation (IUGR) fetal rats [163]. The study reported nicotine-linked reduction in placental cholesterol transporters including scavenger receptor class B type 1 (SR-B1), ABCA1, ABCG1, and liver X receptor (LXR) α and β through nAChR and LXR signaling, respectively, resulting in reduced blood cholesterol levels in female fetal rats [163]. These studies indicate the potential adverse impact of nicotine exposure on placental immune programming and transporters, affecting fetal development.

Though NHP models provide high translational relevance, studies evaluating effects of nicotine on placenta health using NHP models are limited. A study utilizing pregnant rhesus macaques to elucidate the effects of prenatal nicotine exposure on placenta perfusion observed chronic nicotine-induced reduction in fetal blood supply due to decrease in placental volume blood flow [131]. This effect was also paralleled with increase in STBs sprouting and numbers of villous CTB islands, signifying nicotine associated adverse effects on placenta vascular development. Importantly, the same study further reported that maternal Vitamin C supplementation ameliorated these deleterious effects of nicotine on placental hemodynamics, suggesting the consideration of Vitamin C supplementation in pregnancy as a safe treatment option that may help to mitigate toxic effects on fetuses of pregnant women who cannot quit smoking [131].

Overall, animal models significantly contribute to our understanding of how nicotine affects placenta health and subsequently its potential linkage to adverse birth outcomes or to impaired postnatal development. These studies demonstrate that nicotine alone or as a part of cigarette smoke or e-cigarette aerosols can induce deleterious effects on placenta development through several pathobiological mechanisms including, but not limited to, impaired placental blood flow, altered redox signaling, and endocrine pathways. These studies also indicate that the effects of in utero nicotine exposure can depend on product composition, dose, and exposure window. However, currently, the research field lacks animal studies that interrogate combined effects of nicotine or smoke exposures, HIV-1, and ART regimens in pregnancy. This issue is influenced by a broader limitation of the field, which remains in a lack of robust pregnancy rodent models that recapitulate HIV-1 replication, placental transmission, and pregnancy specific maternal-fetal pathobiology. As a result, whether and how HIV-1-ART-nicotine interactions affect placenta, pregnancy outcomes, and transplacental virus transmission remain unknown. Future efforts in developing HIV-1 replication competent rodent models which can breed and recapitulate vertical virus transmission biology as well as in improving standardized inhalation paradigms that can be combined with clinically relevant ART exposure are needed.

## Emerging models

Current research models to study maternal-fetal interface, comprehensively discussed above, have provided critical mechanistic and toxico-pharmacological insights on HIV-1 infection, ART exposure, nicotine or smoking-related injury. However, each platform presents inherent limitations. Trophoblast cell lines lack higher order architecture. Primary trophoblasts have limited lifespan. Human placenta explants preserve tissue context but are not readily scalable. Dual perfusion provides transplacental drug transfer information, but it is restricted largely to term tissue and short experimental timeline. For these reasons, emerging models such as 3D trophoblast organoids, and placenta-on-a-chip are now being considered to address questions that require longer culture duration, defined trophoblast lineage, multicellular interaction, flow dependent transport, and stronger alignment to in vivo placental physiology. Though these models have not been widely used in HIV-1 and associated comorbidity research, they hold a solid potential for addressing HIV-1, ART, and nicotine converge on susceptibility or barrier to infection, oxidative stress, mitochondrial injury, ER stress, angiogenic imbalance, immune activation, and transporter remodeling [164–167]. Thus, broader implementation of these models to advance our understanding of HIV-1 comorbidity–associated effects on placental development is both timely and warranted.

### 3D trophoblast organoids

3D trophoblast organoids are relatively new with the first report in 2018 [168, 169]. To date, few research groups have successfully established 3D trophoblast organoids that are typically generated using purified CTBs from first-trimester placenta [168–170]. However, some reports do consist of iPSCs- or term-placenta-derived 3D trophoblast organoids [171–175]. These 3D models present self-renewing abilities which can differentiate into both STBs and EVTs and closely resemble transcriptomic as well as functional properties of in vivo placentas. Additionally, these organoids can be cultured over a year, allowing long-term assessment of placenta development and function under both healthy and adverse conditions [168, 169, 176]. Of significance to this review article, trophoblast organoids emerge as a relevant model to study placental susceptibility and responses to virus infection or xenobiotic exposures [177–181]. Stem cell–derived trophoblast organoids have revealed selective susceptibility to SARS-CoV-2 and Zika virus infection, resulting in impaired syncytialization [173, 177, 178]. Additionally, these 3D models have revealed trophoblast susceptibility to Oropouche virus, and *Toxoplasma gondii*, indicating potential mechanism of vertical transmission [179, 182]. Yang et al. showed the potential of matched trophoblast and decidual organoids derived from human placentas obtained at late gestation for evaluating the comparative susceptibility and antiviral defenses at the maternal-fetal interface. Researchers observed that decidual organoids are more susceptible to human cytomegalovirus (HCMV) than matched trophoblast organoids. Furthermore, co-culture experiment consisting trophoblast and decidual organoids showed that trophoblast-derived immunomodulatory factors protect decidual cells from HCMV infection, highlighting the importance of these models in studying vertical transmission of pathogens and differences in defense responses by fetal-derived placental trophoblasts and the maternal decidua at the maternal fetal interface [171]. Additionally, some studies have examined the potential of trophoblast organoids to study effects of xenobiotic exposure. Trophoblast organoids derived from first trimester placenta express key ABC transporters at levels comparable to primary villous tissue and were successfully used to model drug-drug interactions involving P-gp, BCRP, MRP1, and MRP2 [180]. Similar trophoblast organoids were employed for evaluating therapeutic rescue of xenobiotic or stress induced placental dysfunction. Recently, Huang et al. employed trophoblast organoids to investigate how low dose of aspirin alleviate preeclampsia. Researchers observed multifactorial effect of aspirin in preventing preeclampsia including restored sFLT-1/PlGF balance, reactivated PI3K-AKT-mTOR signaling, and improved mitochondrial and hormonal metabolism [183]. Furthermore, organoid models were also applied to study responses to environmental toxicants exposure. A study utilized trophoblast organoids to understand how perfluorooctanoic acid (PFOA) or perfluorooctanesulfonate (PFOS) affect placental function. Exposure to PFOA or PFOS inhibited trophoblast differentiation and hormone secretion through cAMP response element-binding protein (CREB) antagonism, providing potential underlying mechanisms for PFOA or PFOS associated low birth weights [184].

Although 3D trophoblast organoids provide benefits over monolayer cell cultures, limitations are recognized. In general, trophoblast organoid systems have inverted polarity [168, 169, 185]. In these systems, the STB-like compartment is oriented inward of the 3D structure. Such structural orientation restricts exposure modeling at the maternal side of the placenta. Recent apical out trophoblast organoid designs addressed these concerns by enabling STB development at the outer surface, thereby improving the physiological relevance of maternal-facing exposure studies [170, 186]. Another important limitation is lineage drift or altered cell state composition during culture [187–189]. This issue is particularly critical for HIV-1, ART, and nicotine exposure studies as changes in trophoblast state may be misinterpreted as toxicity, warranting careful assessment [190, 191]. Overall, 3D trophoblast organoids would allow long-term co-exposure in vitro studies, elucidating effect of combined exposure on placental development and functions including, but not limited to, trophoblast differentiation, endocrine output, transport across the barrier, and stress signaling.

### Placenta-on-a-chip

Placenta-on-a-chip is an emerging in vitro platform for studying drug transfer across the placental barrier under dynamic microenvironment. This new platform based on microfluidic chip devices mimics not only the structure and function of the human placental barrier but also the blood circulation of placenta. In contrast to static trophoblast monolayer culture of traditionally utilized Transwell in vitro model, placenta-on-a-chip provides a dynamic microenvironment consisting of controlled flow, shear stress, extracellular matrix support, and trophoblast-endothelial co-culture. Such multilayered configuration and hemodynamic support enable close recapitulation of structural and functional features of the placental barrier, allowing accurate prediction of drug transport across placenta [192]. A proof-of-concept study successfully implemented the placenta-on-a-chip platform in measuring the transfer of caffeine across the placental barrier [193]. Furthermore, a similar platform was utilized to demonstrate limited maternal-to-fetal transfer of glyburide, a drug prescribed to treat diabetes, due to efflux transporters present on trophoblasts, signifying physiological relevance of the placenta-on-a-chip model [194]. Recent studies have extended this platform to study the effects of nanoparticles and toxicants. These studies found that titanium dioxide and copper oxide nanoparticles impair trophoblast differentiation, hormone secretion, glucose transport, and barrier integrity, and induce inflammatory signaling [195, 196]. Importantly, the placenta-on-a-chip model has been employed to study placental barrier responses and integrity under pathological conditions such as preeclampsia [197] and bacterial infections [198]. Altogether, the placenta-on-a-chip model holds great potential in preclinical developmental toxico-pharmacology studies. The features offered by this model would particularly be important for studying comorbidity influence on transplacental transfer, placenta metabolism, and pathobiology at the maternal-fetal interface.

## Conclusion and future directions

Despite significant advances made using commonly utilized experimental placenta models for improving our understanding of potential toxico-pharmacological effects of HIV-1, ART, or nicotine on pregnancy outcomes, there is a knowledge gap about whether and how the complex interactions among HIV-1, ART, and nicotine exacerbate the adverse effects on placental biology affecting pregnancy outcomes. For effectively determining these comorbid effects, more efforts are needed towards developing physiologically robust in vitro and rodent models that recapitulate HIV-1 replication, transplacental transmission, and pregnancy specific maternal-fetal pathobiology, and additionally, enable high-throughput assessments. Future efforts in developing HIV-1 replication competent rodent models that support breeding and recapitulate mechanisms of vertical virus transmission are warranted. Furthermore, advances in in vitro models are needed which include but may not be limited to development of vascularized and immune competent 3D organoids and multiorgan-on-a-chip platforms like placenta-brain. These would improve physiological relevance of models through boosting crosstalk among immune cells, trophoblasts, and endothelial cells in a multicellular microenvironment supported by vasculature, accurately predicting fetal exposure and safety. Overall, more than a million infants are born to PWLWH. Thus, it is timely to understand any potential adverse effects on the placenta linked to HIV-1 and associated comorbidities in order to offer effective care to PWLWH and their fetuses. Due to ethical concerns and practical challenges associated with obtaining human placenta samples, experimental placenta models remain essential to uncover unknown adverse effects and linked potential mechanisms underlying compromised pregnancy outcomes. Thus, continued research efforts in developing physiologically relevant experimental placental models are critically needed for improving our understanding of role of placenta deficits induced by HIV-1 and associated comorbidities in adverse pregnancy outcomes and related mechanistic pathways, and for developing prevention as well as intervention strategies.
